# Explicit and implicit attitudes toward sustainability in outdoor athletes

**DOI:** 10.3389/fpsyg.2025.1708079

**Published:** 2026-01-05

**Authors:** Sabine Hoja, Petra Jansen

**Affiliations:** Institute of Sport Science, Faculty of Human Sciences, University of Regensburg, Regensburg, Germany

**Keywords:** pro-environmental attitudes, outdoor sports, team and individual sports, explicit and implicit attitudes, implicit association test (IAT)

## Abstract

Understanding athletes' attitudes toward sustainability is essential, as sports can significantly influence environmental awareness and behavior. Differences may exist between athletes who practice individually and those who compete in teams, yet little is known about the underlying nature of these attitudes-especially when considering implicit dimensions. The present study builds on the former research by investigating whether individual and team outdoor athletes differ in both their explicit and implicit sustainable attitudes. One hundred athletes participated, with 45 from individual sports and 55 from team sports. Participants completed an explicit rating task, the Implicit Association Test, the Connectedness to Nature Scale, and a demographic questionnaire. Results indicate that individual outdoor athletes rated non-sustainable concepts as less positive than team outdoor athletes suggesting more sustainable attitudes. No differences emerged between the two groups regarding implicit attitudes and explicit attitudes toward sustainable concepts. These findings suggest that individual and team athletes may differ in their explicit evaluations of unsustainable concepts, while implicit attitudes appear more similar. This highlights the need to investigate the underlying processes in more depth.

## 1 Introduction

Several individual factors are related to sustainable consumption or pro-environmental behavior, such as values, attribution of responsibility, awareness of consequences, personal and social norms, attitudes, intentions, perceived behavioral control, and habits, which are described in the comprehensive action determination model (Klöckner, 2013). However, all these factors cannot fully explain sustainable consumption behavior, so theoretical assumptions argue for consumer activation at a deeper level (Thiermann and Sheate, 2021). Thiermann and Sheate (2021) conceptualized a model with a relational path as a separate path alongside the normative path in the Comprehensive Action Determination Model, which determines environmental behavior independently of other predictors. This relational pathway includes the concepts of connectedness, empathy, and compassion for nature. This is in line with Wamsler et al. (2021) model that transformative qualities like values, connectedness, and empathy are related to pro-environment behavior. This model was elaborated by Stenfors et al. (2025), who present the relationships between meditation practice and transformative qualities, including nature connection, mental wellbeing, and pro-environmental behavior. Nature connectedness's critical role for pro-environmental behavior is always mentioned in all these models and previous research (Barragan-Jason et al., 2022). Connectedness to nature, which can be defined as “an individual's affective, experiential connection to nature” (Mayer and Frantz, 2004, p. 504), was one of the relevant predictors for sustainable attitudes and behavior.

In sports science, outdoor athletes might have a higher natural connectedness than indoor athletes. One study investigated whether athletes of various sports differ in their sustainable attitudes and behavior (Jansen et al., 2024a). The results showed that nature connectedness is one of the relevant predictors of sustainable attitudes and behavior. Furthermore, athletes from outdoor sports are more connected to nature than athletes from indoor sports, and athletes from individual outdoor sports show the highest values in sustainable attitudes and behavior. Nevertheless, in this study, only explicit attitudes were investigated and measured with direct questions.

For this, this research project aims to examine attitudes toward sustainability in more detail. Explicit attitudes are only one type of attitude. In dual process models, it is assumed that human attitudes always consist of controlled or reflective and uncontrolled or intuitive aspects (Bellini-Leite, 2022). The controlled aspects (explicit attitudes) can be measured with direct questions, and the uncontrolled aspects (implicit attitudes) require indirect measures, such as the Implicit Association Test (IAT).

Explicit attitudes are based on deliberate thinking and can be measured with self-report methods, such as asking participants to rate their agreement with statements on Likert scales (Panzone et al., 2016). In contrast, implicit attitudes operate largely outside of conscious awareness and influence behavior through automatic, unconscious processes (Devos, 2008). Explicit and implicit measurement methods differ substantially because unconscious aspects of attitudes cannot be captured with standard questionnaires. Instead, indirect measures such as the Implicit Association Test (IAT; Greenwald et al., 1998) are required, where participants do not consciously deliberate on their responses. In the IAT, participants rapidly classify stimuli–typically words or pictures–into their respective categories by pressing the corresponding keys. The test is based on the principle that individuals respond faster and more accurately when two strongly associated concepts are paired, and slower when the concepts are less closely related. In this way, the IAT provides an indirect measure of association strength, without asking participants to explicitly report it (Greenwald et al., 2022).

To summarize, previous research from Germany has shown that athletes from individual outdoor sports report higher sustainable explicit attitudes when using questionnaires than athletes from team outdoor sports (Jansen et al., 2024a). In this study, we wanted to expand on the former study and elaborate on the former results. We wanted to investigate whether explicit and implicit attitudes differ between individual and team outdoor sports. A possible reason might be that individual outdoor sports athletes are more affected by a dynamic interplay between athletes and elemental aspects of the environment, such as wind (Jansen et al., 2024a; Melo et al., 2020). Hypotheses

(1) Individual outdoor sport athletes will show more positive explicit attitudes (measured with a rating procedure) and more positive implicit attitudes (measured with the IAT) toward sustainability compared to team outdoor athletes.
(2) Explicit and implicit attitudes toward sustainability can be predicted by certain factors, including type of sport, age, and connectedness to nature.

## 2 Methods

### 2.1 Study design

The study employs a correlational and quasi-experimental design.

### 2.2 Study sample

#### 2.2.1 Participants

The study sample consisted of 100 outdoor athletes (64 men, 32 women, and one non-binary individual; gender information was missing for three participants). Participants had to be at least 18 years old, have practiced their sport for more than 8 years, and train at least twice per week. The 45 individual athletes represented athletics (1), climbing (1), cycling (8), equestrian (2), golf (5), gymnastics (1), mountain biking (3), running (10), skiing (3), snowboarding (2), strength training (1), swimming (2), tennis (3), and triathlon (3). The 55 team athletes, in contrast, represented American football (2), cycling (1), fistball (1), soccer (48), handball (2), and volleyball (1). For further details on the athletes, see Table 1.

**Table 1:** Participant demographics.

**Type of sport**	**Gender**	**Age^a^** **(years)**	**Sports experience (years)**	**Weekly training (hours)**	**Club membership** ^**b**^		**Participation in competitions** ^**b**^	
		Mean ± SD	Mean ± SD	Mean ± SD	Yes (%)	No (%)	Yes (%)	No (%)
Individual (*n* = 45)	Male: 22 Female: 20 Non-binary: 1 No answer: 2	32.27 ± 14.804	16.689 ± 8.793	6.489 ± 3.628	51.1	46.7	60.0	37.8
Team (*n* = 55)	Male: 42 Female: 12non-Binary: 0 no answer: 1	23.40 ± 4.744	16.545 ± 4.131	6.000 ± 1.895	94.5	3.6	98.2	0.0

Percentages do not add up to exactly 100% due to a few missing responses. ^1^Significant difference between team and individual sports athletes (*t*-test). ^2^Significant difference between team and individual sports athletes (Chi-square test).

#### 2.2.2 Sample size calculation

For the first hypothesis, two independent *t*-tests were planned, one for each dependent variable (explicit attitudes toward sustainability and implicit attitudes toward sustainability), with the independent factor being the type of sport (individual sport vs. team sport). The effect size for sustainable attitudes between individual and team outdoor athletes in the study by Jansen et al. (2024a) was large (d = 0.811). However, as we expect smaller effects for implicit attitudes, we assume a medium effect size of d = 0.66 for the present study. Due to multiple testing (two independent *t*-tests), the significance level was set to α = 0.025. With an alpha level of.025, a power of 1–β = 0.80, and an expected effect size of d = 0.66, a total of *N* = 90 participants were required, with 45 in each group (individual sports and team sports).

For the second hypothesis, three predictors (type of sport, age, and connectedness to nature) were intended to be examined for their relation to the two dependent variables (explicit attitudes toward sustainability and implicit attitudes toward sustainability). Due to multiple testing and the use of two regression analyses, the significance level was set to α = 0.025. A power analysis for linear regression (Faul et al., 2007) with a medium effect size of *f*^2^ = 0.15, an alpha level of.025, and a power of 1–β = 0.80 indicated that *N* = 91 participants were required for each regression analysis.

### 2.3 Procedure

The online experiment was implemented in OpenSesame (Mathôt et al., 2012) and conducted on JATOS (Just Another Tool for Online Studies). The study link was distributed to athletes via newsletters and social media between January and April 2025. All participants first provided informed consent. Subsequently, they completed the tasks and questionnaires in the following order: explicit attitudes toward sustainability, implicit attitudes toward sustainability, the Connectedness to Nature Scale, and the demographic questionnaire.

### 2.4 Material

#### 2.4.1 Demographic questionnaire

The demographic questionnaire assessed the following variables: sex (male, female, non-binary), age, education level (categorical: no qualification, high school, A-levels, bachelor, master, PhD), net monthly income (in euros), type of sport, years of experience in the primary sport, weekly training hours in the primary sport, club membership (yes/no), participation in competitions (yes/no), and sport-related salary (yes/no).

#### 2.4.2 Explicit attitudes toward sustainability

For the explicit rating task, five words related to sustainability (bicycle, second-hand clothing, reusable bag, vegetables, and solar energy) and five words unrelated to sustainability (cruise ship, fast fashion, single-use plastic, sausage, and coal power plant) were selected from the study by Winkelmair et al. (2023). Thirty-two participants had rated the words in terms of their perceived sustainability prior to their use in the study by Winkelmair et al. (2023). The task consisted of the following question: “*Please indicate on a scale from 1 to 7 (1* = *negative, 7* = *positive) how you feel about the following terms/items.”* Participants had 5 s to respond in order to elicit a spontaneous reaction. For analysis, the mean rating was calculated separately for the sustainable and unsustainable terms.

#### 2.4.3 Implicit attitudes toward sustainability

**Figure 1:**
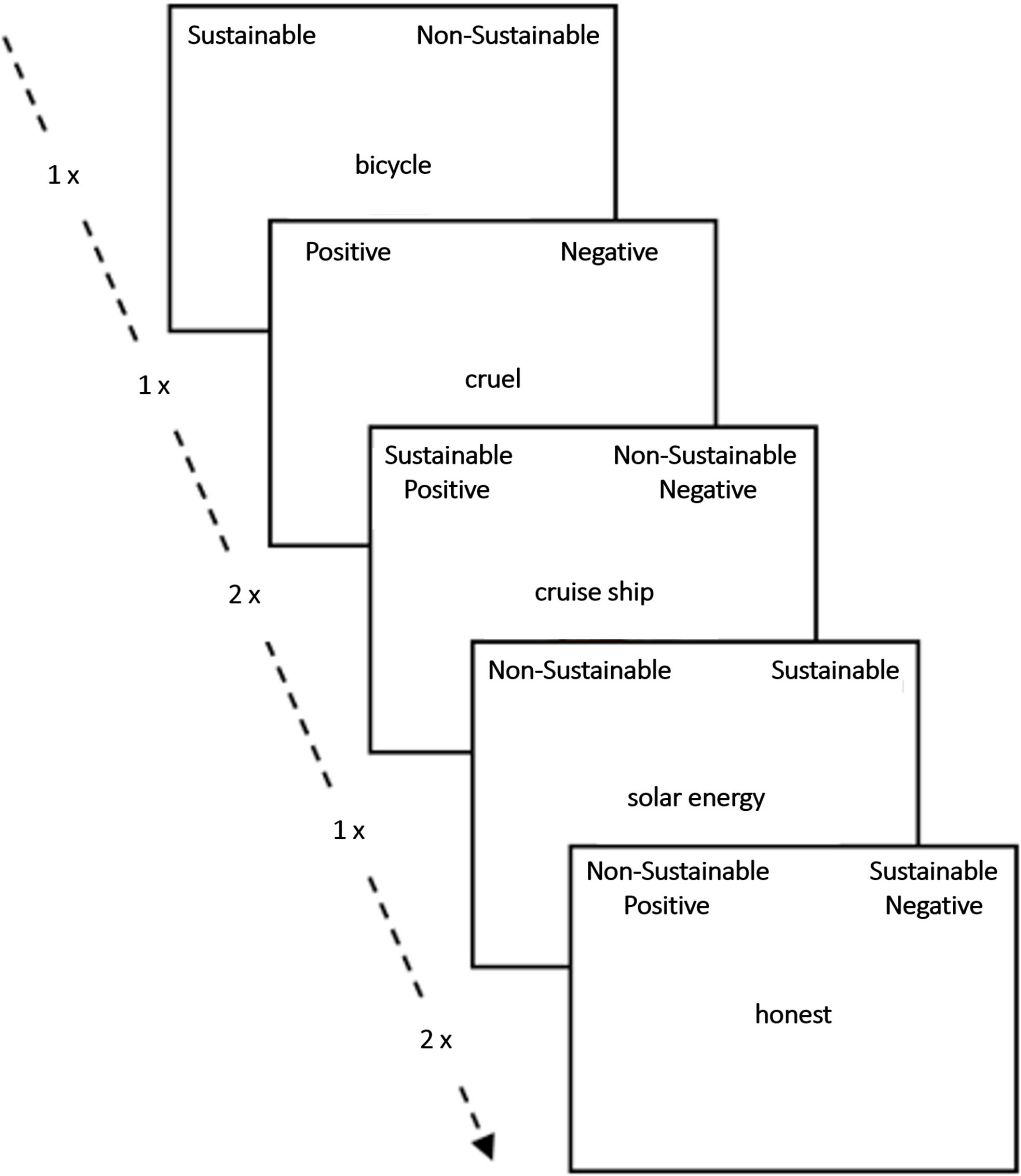
Illustration of the Implicit Association Test (IAT) procedure. The IAT measures the strength of associations between concepts (sustainable, unsustainable) and evaluations (e.g., good, bad) or stereotypes (positive, negative). The main idea is that responding is easier when closely related items share the same response key. A detailed description can be found in the methods section, lines 142-163.

The standard Implicit Association Test (IAT; Greenwald et al., 1998) assesses implicit attitudes. The task comprises four categories–two target categories and two attribute categories–and corresponding stimulus words. The target categories were “sustainable” and “not sustainable”, and the attribute categories were “positive” and “negative”. As stimuli, the same 10 terms (bicyle, second-hand clothing, cruise ship etc.) as in the explicit evaluation were used as target words, along with five positive words (popular, joyful, valuable, honest, and sunny) and five negative words (unloving, cruel, heartless, sad, and lonely) from the Berlin Affective Word List (Võ et al., 2009). In each trial, a stimulus was presented in the center of the screen and participants were instructed to categorize it by pressing “D” for the category displayed on the left or “K” for the category displayed on the right. In Blocks 1 and 5, participants categorized only target words into the target categories (“sustainable” vs. “not sustainable”), which were displayed in black font in the upper left and right corners of the screen. In Block 2, attribute words were categorized into the attribute categories (“positive” vs. “negative”), which were displayed in green font in the same locations. Blocks 3, 4, 6, and 7 were combined blocks, with one target category (black font) and one attribute category (green font) presented on each side. Target words appeared in odd-numbered trials and attribute words in even-numbered trials. Each stimulus belonged to only one category and had to be categorized accordingly. If an incorrect response was given, a red cross appeared below the stimulus and the program waited until the correct key was pressed. The positions of the target and attribute categories were randomized across participants. In Block 5, the positions of the target categories were switched and remained in that configuration for the subsequent blocks. Figure 1 provides an overview of the entire IAT procedure. For analysis, the D-Score was calculated following the procedure of Greenwald et al. (2022). Higher IAT scores indicate an implicit preference for sustainable items.

#### 2.4.4 Connectedness to Nature Scale (CNS)

Connectedness to nature was measured with 13 items rated on a 5-point Likert scale ranging from 1 (strongly disagree) to 5 (strongly agree) (Pasca et al., 2017). An example item is: “*Like a tree can be part of a forest, I feel embedded within the broader natural world.”* Reliability could not be assessed because 40 % of the participants did not complete the questionnaire.

#### 2.4.5 Deviation from preregistration

The Connectedness to Nature Scale was intended to be one predictor in the regression analyses during preregistration. However, nearly 40 % of participants did not complete the questionnaire, so the scale could not be included. Consequently, we incorporated all other socio-demographic variables (sport type, gender, age, sport experience, weekly trained hours, club membership, participation in competition) in further exploratory, non-preregistered analyses to gain more insight into the results.

## 3 Results

### 3.1 Attitudes toward sustainable terms

**Figure 2:**
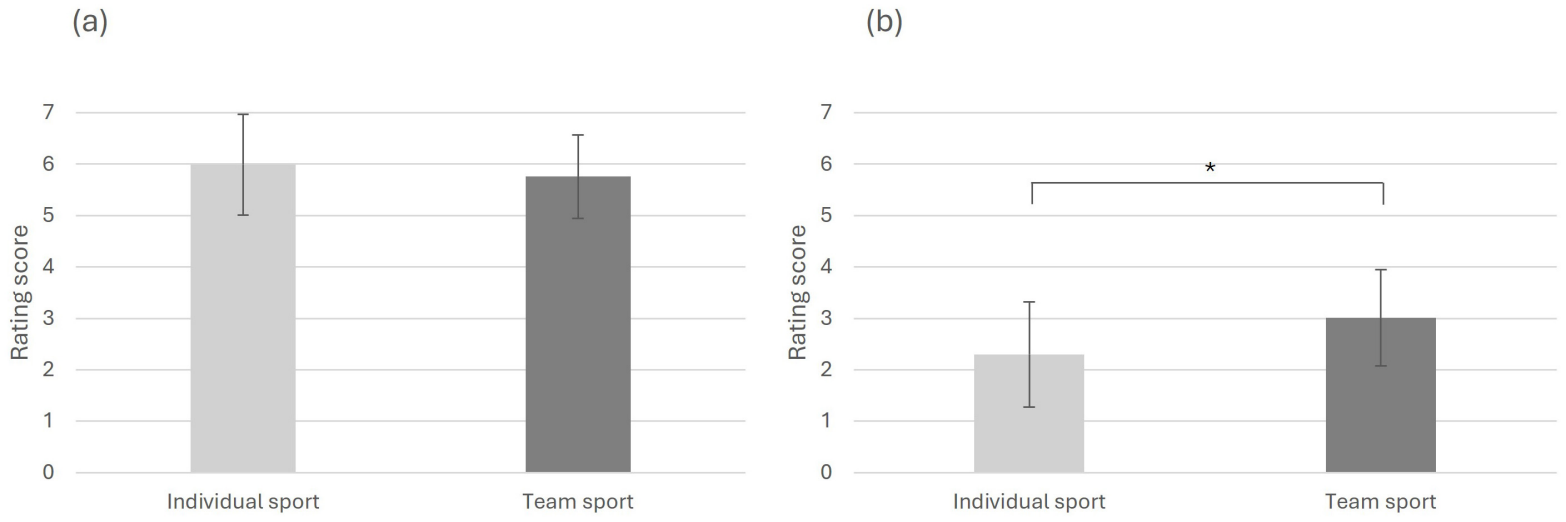
Explicit attitudes toward sustainability. **(a)** Sustainable terms; **(b)** Unsustainable terms. *p < 0.05.

There was no significant difference between athletes from individual sports (M = 5.989, SD = 0.980) and athletes from team sports (M = 5.759, SD = 0.812) in explicit attitudes toward sustainable terms, *t* (98) = 1.283, *p* = 0.101, *d* = 0.26, 95% CI [−0.126, 0.585], see Figure 2a.

### 3.2 Attitudes toward unsustainable terms

However, there was a significant difference between athletes from individual sports (M = 2.302, SD = 1.023) and athletes from team sports (M = 3.011, SD = 0.941) regarding explicit attitudes toward unsustainable terms, *t* (98) = −3.603, *p* < 0.001, *d* = −0.72, 95% CI [−1.099, −0.318], see Figure 2b.

### 3.3 Implicit attitudes

**Figure 3:**
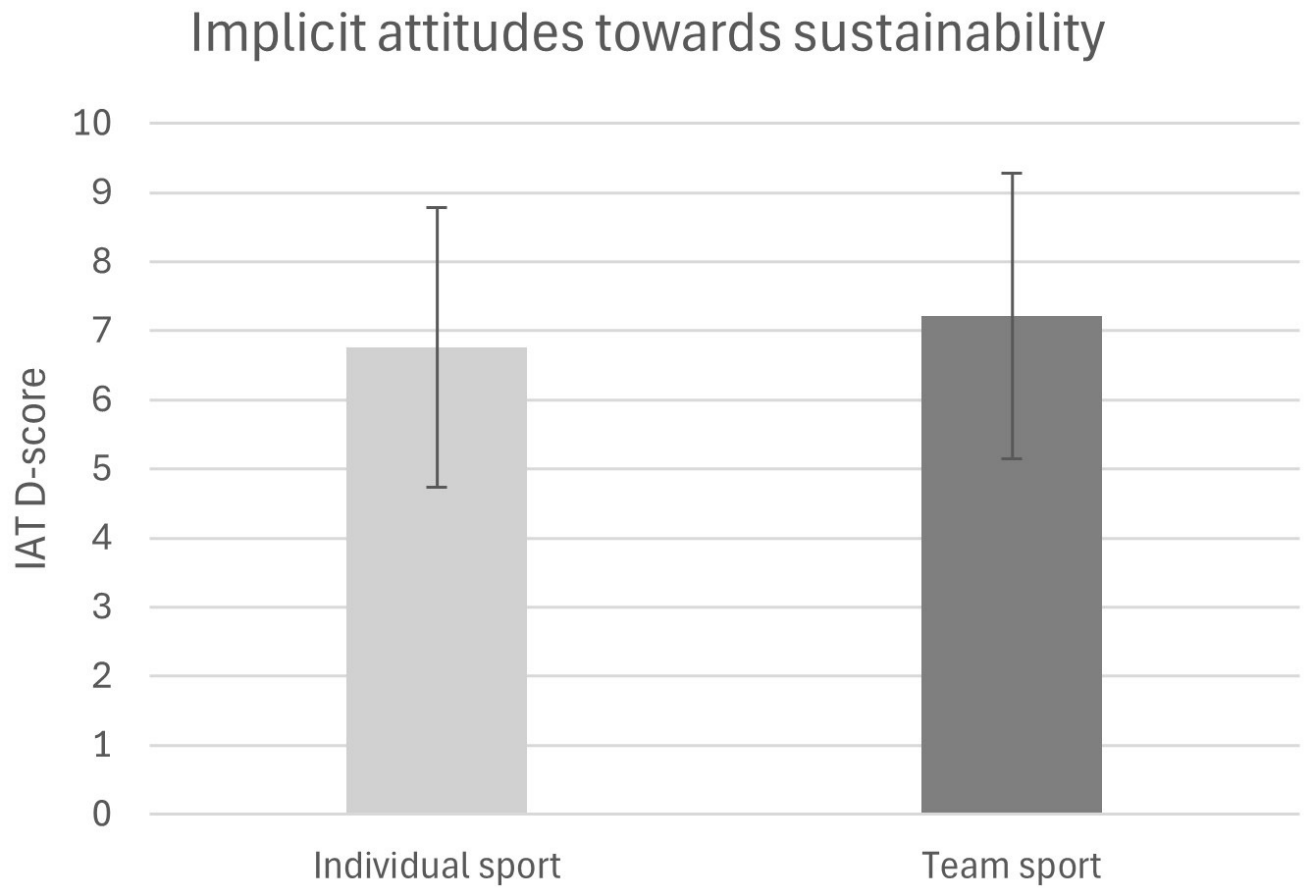
Implicit attitudes toward sustainability.

Analysis of implicit attitudes toward sustainability revealed no significant difference between athletes from individual sports (M = 6.756, SD = 2.025) and athletes from team sports (M = 7.210, SD = 2.062), *t* (98) = −1.103, *p* = 0.136, *d* = −0.22, 95% CI [−1.269, 0.362], see Figure 3.

### 3.4 Regression analyses

The regression models were significant for explicit attitudes toward unsustainable terms, *F*_(7, 93)_ = 4.06, *p* < 0.001, *R*^2^ = 0.25 and implicit attitudes, *F*_(7, 93)_ = 2.17, *p* = 0.045, *R*^2^ = 0.15, but not for explicit attitudes toward sustainable terms, *F*_(7, 93)_ = 1.46, *p* =0.194, *R*^2^ = 0.11.

For explicit attitudes toward non-sustainable items, type of sport (β = 0.60, *p* = 0.036), club membership (β = −0.56, *p* = 0.038), and competition participation (β = 0.89, *p* = 0.016) were significant predictors. Team-sports athletes rated unsustainable items more positive than individual athletes, Club membership and non-participation in competition lead to more positive attitudes toward unsustainable terms.

For implicit attitudes, type of sport (β = 1.18, *p* = 0.044), sport years (β = −0.002, *p* = 0.031), and competition participation (β = −1.89, *p* = 0.011) emerged as significant predictors. Team-sports athletes rated sustainable items implicitly more positive than individual athletes. Participation in competition and fewer sports years lead to more positive implicit attitudes toward sustainable terms.

## 4 Discussion

The results obtained in a previous study (Jansen et al., 2024a) were partially replicated, specifically for explicit attitudes toward unsustainable terms. Individual-sport athletes rated the presented terms as less positive than team-sport athletes. One reason might be that the contact with nature in individual sports athletes differs from that of team athletes. Many outdoor team athletes came from sports such as soccer, which does not involve a dynamic interaction between athletes and components of nature (Melo et al., 2020). For this, the nature connectedness might be higher for the individual outdoor athletes. Unfortunately, we could not measure this because only 40% answered the nature connectedness questionnaire.

There was no significant difference between the groups (individual-sport athletes and team-sport athletes) in their attitudes toward sustainable terms. It is also possible that, for the obviously positive, sustainable terms, participants' responses were influenced by social desirability.

This hints that it is not the type of sport but other factors that are likely influential, like a structural environment. Exploratory analyses showed that club membership and competition participation are relevant variables in explaining explicit attitudes toward unsustainable terms, maybe more than the type of sport. During a club membership, the members might experience social facilitation and group cohesion, which are irrelevant in competition sports (Zhu and Han, 2019). Those prosocial traits are related to sustainable attitudes (Jansen et al., 2024b).

At first glance, no significant difference was observed between athletes from individual and team sports for implicit attitudes, which capture more automatic responses. In a second analysis, the type of sport, competition participation, and the year of practicing the sport are related to implicit attitudes. This showed that implicit attitudes on sustainability in athletes depend on many things and need further investigations, because both components of attitudes, explicit and implicit ones are important in explaining sustainable behavior. Because the Connectedness to Nature Scale could not be included in the regression analyses, no conclusions can be drawn regarding whether a certain level of connectedness to nature might predict more sustainable attitudes. Socio-demographic variables like socio-economic status were also not analyzed, though it is essential for sustainable behavior (Gladstone and Bellezza, 2025).

### 4.1 Limitations and future research

The group of team-sport athletes consisted almost exclusively of soccer players, which limits the generalizability of the results. Greater attention should have been paid to recruiting athletes from a broader range of outdoor team sports. Outdoor space can differ in several ways between different sports, like the forest or only outside on the soccer field. Also, the differentiation between individual and team sports is challenging because there are at least six typologies that distinguish types of sports groups according to levels of structural interdependence (Evans et al., 2012). A further limitation is that the connectedness to nature scale could not be analyzed because of 60% random missing data. The missing data were unexpected and hint that the order of the questionnaires and the experimental setting should be considered carefully.

Furthermore, investigating actual sustainable behaviors (e.g., consumer and mobility choices) in athletes and their relationship with nature connectedness rather than solely relying on rating tasks could help better understand the relationship between attitudes and behavior in athletes from different disciplines.

## Data Availability

The raw data supporting the conclusions of this article are available under https://osf.io/sm78c?view_only=3ed71dc20097455d820142c0a27aa378.
